# Supplementary material for: Death of an offspring and parental risk of ischemic heart diseases: A population-based cohort study
Source: PLoS Med. 2021 Sep 29;18(9):e1003790. doi: 10.1371/journal.pmed.1003790 (PMC8480908; doi:10.1371/journal.pmed.1003790)
Supplement: S1 Analysis plan — AMI, acute myocardial infarction; IHD, ischemic heart disease. (DOCX) [file pmed.1003790.s002.docx]

**S1 Analysis plan: Parental risk of acute myocardial infarction and ischemic heart disease after the death of a child**

- **Research question**

Is there an association between death of a child and the risk of incident acute myocardial infarction (AMI) and ischemic heart disease (IHD)?

- **Study population**

The study participants are the parents of live-born children during 1973-2016 in the Danish Medical Birth Register and all births during 1973-2014 in the Swedish Medical Birth Register. The index parents are identified through linkage to the births (in the Danish Civil Registration System and the Swedish Multi-Generation Register) and will be excluded if the persons had a history of IHD. The follow-up will start on January 1, 1978 in Denmark and January 1, 1987 in Sweden if the index person had at least one live child, otherwise on the birthdate of first child (in Denmark or Sweden) or the date of immigration to Denmark/Sweden if they immigrated with their child(ren). Follow-up ends at the first diagnosis of IHD or AMI respectively, emigration, death, or the end of study period (December 31, 2016 in Denmark and December 31, 2014 in Sweden), whichever came first.

- **Exposure**

Death of a child. The exposure will be regarded as time-dependent variable. An exposed person will contribute to the unexposed group from the start of follow-up to the date of death of a child and to the exposed group afterwards. To better understand causality, we will further perform analyses with exposure categorized according to the causes of child’s death (e.g., cardiovascular diseases (CVDs), other natural and unnatural) through the International Classification of Diseases (ICD) codes, age at loss (e.g. <1, 2-5, 6-12, 13-18 and >18) and number of live children at loss (e.g. 0, 1-2 and 3 or more).

***The ICD-codes used to classify causes of death***

| Cause of death | Denmark | Sweden |
| --- | --- | --- |
| Death due to CVDs | ICD-8: 390-458  ICD-10: I00-I99 | ICD-8: 390-458  ICD-9: 390-459  ICD-10: I00-I99 |
| Unnatural death | ICD-8: 7959, 79621, E800-E999  ICD-10: R95, R96, R98, V01-Y98 | ICD-8: 7959, 79621, E800-E999  ICD-9: 798, E800-E999  ICD-10: R95, R96, R98, V01-Y98 |
| Death due to other natural causes | The rest of the ICD codes | The rest of the ICD codes |

CVD=cardiovascular disease; ICD= International Classification of Diseases.

- **Outcomes**

We will identify IHD and AMI through the main diagnosis or the underlying cause of death from the National Hospital Register and the Civil Registration System in Denmark and from the Patient Register and the Cause of Death Register in Sweden using the following ICD codes.

Acute myocardial infarction:

- ICD-8: 410
- ICD-9: 410
- ICD-10: I21, I22

Ischemic heart disease:

- ICD-8: 410-414
- ICD-9: 410-414
- ICD-10: I20-I25
- **Covariates**

The information on the following covariates will be collected from several nationwide registries in Denmark and Sweden: age, sex, country of birth, marital status, education, income, history of hypertension and diabetes before pregnancy at baseline, maternal obesity and smoking in early pregnancy before baseline, personal history of psychiatric disorders, as well as personal and family history of CVDs.

- **Statistical analysis**

Poisson regression will be used to evaluate the association between the death of a child and incident AMI and IHD. The follow-up will be split by age (every five years) and calendar year (every ten years). We will perform analyses with any loss and exposure categorized according to the cause of death (cardiovascular, other natural and unnatural deaths), age at the loss (≤1, 2-5, 6-12, 13-18, and >18) and the number of live children at loss (0, 1-2 and ≥3). We will run both age-adjusted models and multivariable models by adjusting for age and calendar year at follow-up, sex, country, highest education, personal history of psychiatric disorders and CVDs.

In sensitivity analyses we will adjust for marital status, income, parents’ history of CVDs, siblings’ history of CVDs, maternal hypertension and diabetes before and during pregnancy, as well as maternal smoking and obesity in early pregnancy; we will restrict these analyses to those with data on these covariates. A further sensitivity analysis aims to evaluate whether there is change in the risk estimate after excluding individuals who lost a child before baseline.

To test whether the association between death of a child and outcomes of interest differs by gender, age (<50 vs. >=50) and educational attainment, we will conduct stratified analyses and formal tests of interaction with these variables.

***Note:***

*Suggestions for a number of further analyses after the start of the study were included after discussions with coauthors or based on suggestions from editors and reviewers:*

- *During discussions while working on another manuscript one of the co-authors suggested investigating short and long-term effects after bereavement; such analyses were deemed important also for this study and were included also to the analyses of this manuscript.*
- *During the revision of the first draft of the analyses one of the coauthors also suggested to perform case-crossover analyses to explore in more depth the short-term effects observed in the cohort analyses.*
- *Editors and one of the reviewers suggested including p-values for the comparison between exposed and unexposed; we thus performed Student t-tests in case of continuous variables and chi-square test for in case of categorical variables.*
- *As suggested by one reviewer, we conducted a maximally adjusted model by adjusting for marital status, income, parents’ history of cardiovascular diseases, and siblings’ history of cardiovascular diseases in addition to variables in our multivariable model among those without missing data in any variables.*
- *We included analyses with multiple imputation based on suggestions from one of the reviewers.*
