# Supplementary figures and images for: Death of an offspring and parental risk of ischemic heart diseases: A population-based cohort study
Source: PLoS Med. 2021 Sep 29;18(9):e1003790. doi: 10.1371/journal.pmed.1003790 (PMC8480908; doi:10.1371/journal.pmed.1003790)

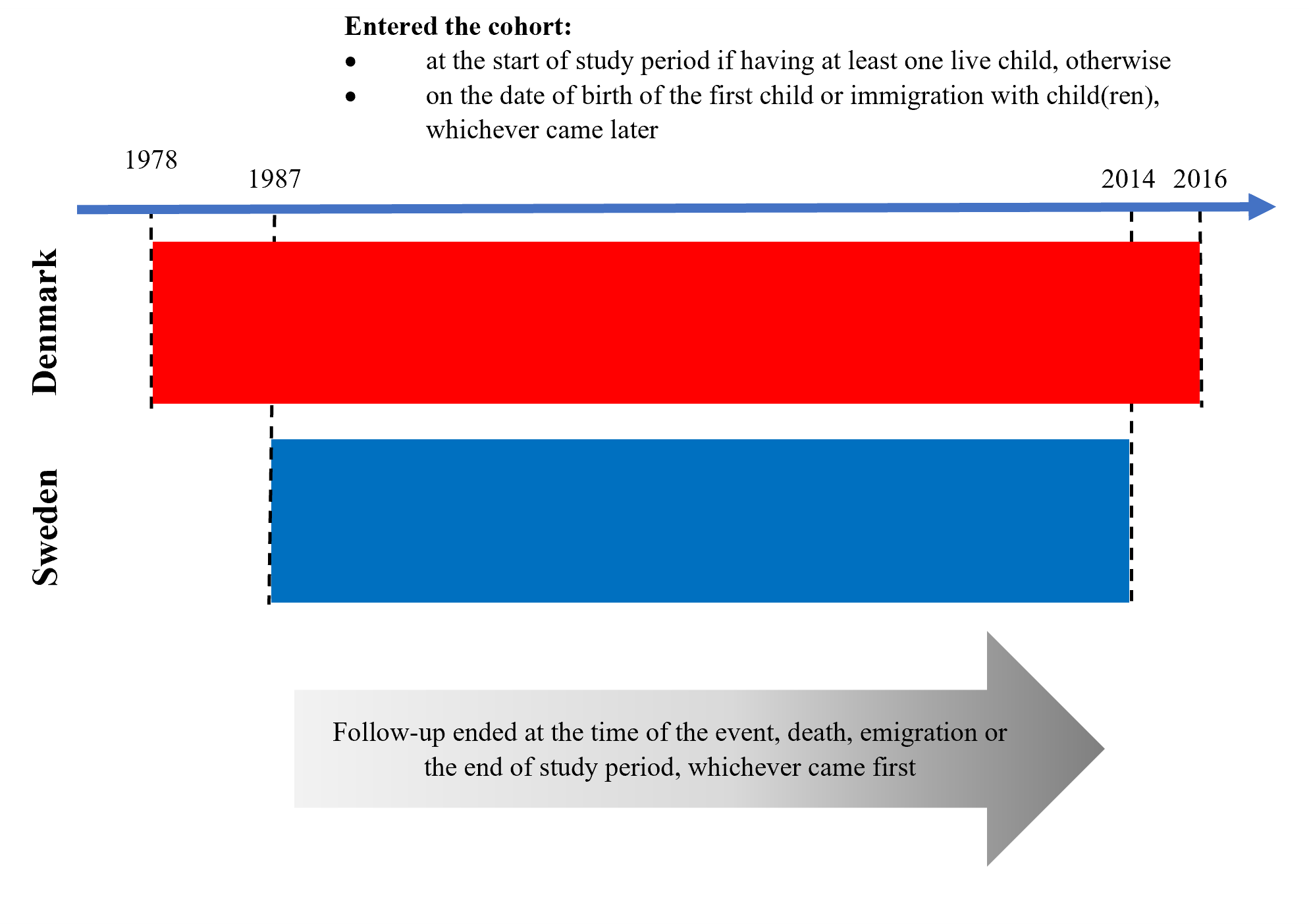

Supplement: S1 Fig — (TIF) [file pmed.1003790.s003.tif]

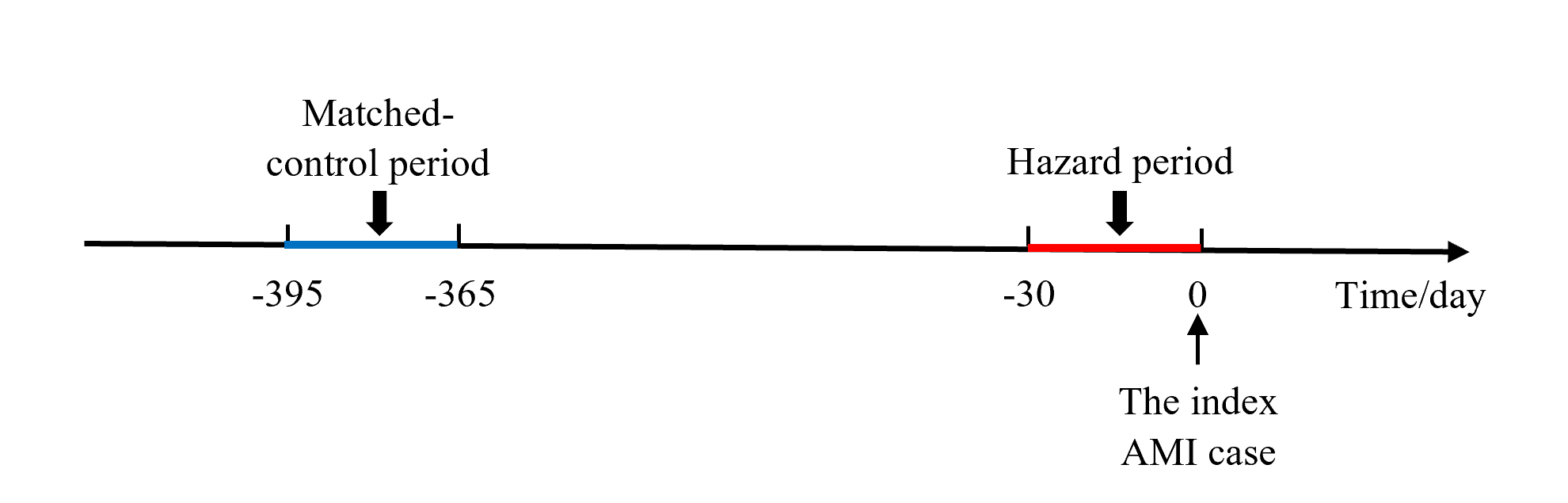

Supplement: S2 Fig — AMI, acute myocardial infarction. (TIF) [file pmed.1003790.s004.tif]
