# Supplementary material for: Death of an offspring and parental risk of ischemic heart diseases: A population-based cohort study
Source: PLoS Med. 2021 Sep 29;18(9):e1003790. doi: 10.1371/journal.pmed.1003790 (PMC8480908; doi:10.1371/journal.pmed.1003790)
Supplement: S1 Table — (DOCX) [file pmed.1003790.s005.docx]

**S1 Table. Population-based registers used to retrieve information for the study**

| **Register** | **Information** | **Period covered** |
| --- | --- | --- |
| Danish Medical Birth Register | Child’s birth date and sex | 1973-2016 |
|  | Maternal smoking in early pregnancy | 1991-2016 |
|  | Maternal body mass index in early pregnancy | 2003-2016 |
| Danish Civil Registration System | Date and cause of death, age, sex, country of birth, linkage to family members, date of migration | 1970-2016 |
|  | Marital status | 1972-2016 |
| Danish Hospital Register | Information on inpatient and outpatient care (diagnosis, date) | Inpatient care: 1977-2016  Outpatient care and emergency department visits: 1995-2016 |
| Danish Central Psychiatric Register | Information on inpatient and outpatient care for psychiatric disorders (diagnosis, date)⃰ | Inpatient care: 1969-2016  Outpatient care and emergency department visits: 1995-2016 |
| Danish Integrated Database for Longitudinal Labor Market Research | Income | 1980-2015 |
|  | Education | 1980-2016 |
| Swedish Medical Birth Register | Child’s birth date and sex, pregestational and gestational hypertension and diabetes | 1973-2014 |
|  | Maternal smoking in early pregnancy | 1982-2014 |
|  | Maternal body mass index in early pregnancy | 1982-1989, 1992-2014 |
| Swedish Multi-Generation Register | Linkage to relatives | 1961-2014† |
| Swedish Total Population Register | Sex, birth year, marital status, country of birth, date of migration | 1973-2014 |
| Swedish Cause of Death Register | Date and cause of death | 1952-2014 |
| Swedish Patient Register | Information on inpatient and specialized outpatient care (diagnosis, date) | Inpatient care: 1969-2014  Hospital-based outpatient care: 2001-2014‡ |
| Swedish Education Register | Education | 1990-2014 |
| Swedish Register of Incomes and Taxes | Income | 1972-2014 |

*All psychiatric inpatient, outpatient and emergency department contacts in Denmark have been reported to the National Hospital Register since 1995.

†Individuals born in 1932 or later and alive on January 1, 1961 or registered later in Sweden are included as index persons in the Multi-Generation Register.

‡The coverage of hospital-based outpatient care is approximately 80%.
