# Supplementary material for: Death of an offspring and parental risk of ischemic heart diseases: A population-based cohort study
Source: PLoS Med. 2021 Sep 29;18(9):e1003790. doi: 10.1371/journal.pmed.1003790 (PMC8480908; doi:10.1371/journal.pmed.1003790)
Supplement: S2 Table — (DOCX) [file pmed.1003790.s006.docx]

**S2 Table. The International Classification of Diseases codes used to identify the diagnoses and the causes of death**

| **Diseases** | **ICD codes** | |
| --- | --- | --- |
|  | **Danish registers** | **Swedish registers** |
| **Medical condition** | | |
| Psychiatric disorders | ICD-8: 290-315  ICD-10: F00-F99 | ICD-8: 290-315  ICD-9: 290-319  ICD-10: F00-F99 |
| Hypertension | ICD-8: 400-404, 63700, 63703, 63704, 63709, 63719  ICD-10: I10-I15, O10, O11, O13-O16 | ICD-8: 400-404, 63701, 63703, 63704, 63709, 63710  ICD-9: 401-405, 642  ICD-10: I10-I15, O10, O11, O13- O16 |
| Diabetes | ICD-8: 249, 250  ICD-10: E10-E14, O24 | ICD-8: 250  ICD-9: 250, 648A  ICD-10: E10-E14, O24 |
| CVD | ICD-8: 390-458  ICD-10: I00-I99 | ICD-8: 390-458  ICD-9: 390-459  ICD-10: I00-I99 |
| Acute myocardial infarction | ICD-8: 410  ICD-10: I21, I22 | ICD-8: 410  ICD-9: 410  ICD-10: I21, I22 |
| Ischemic heart disease | ICD-8: 410-414  ICD-10: I20-I25 | ICD-8: 410-414  ICD-9: 410-414  ICD-10: I20-I25 |
| **Cause of death** | | |
| Death due to CVD | ICD-8: 390-458  ICD-10: I00-I99 | ICD-9: 390-459  ICD-10: I00-I99 |
| Unnatural death | ICD-8: 7959, 79621, 800-999, E800-E999  ICD-10: R95, R96, R98, V01-Y98 | ICD-9: 798, 800-999, E800-E999  ICD-10: R95, R96, R98, V01-Y98 |
| Death due to acute myocardial infarction | ICD-8: 410  ICD-10: I21, I22 | ICD-9: 410  ICD-10: I21, I22 |
| Death due to ischemic heart diseases | ICD-8: 410-414  ICD-10: I20-I25 | ICD-9: 410-414  ICD-10: I20-I25 |

CVD=cardiovascular diseases; ICD=International Classification of Diseases
