# Supplementary material for: Death of an offspring and parental risk of ischemic heart diseases: A population-based cohort study
Source: PLoS Med. 2021 Sep 29;18(9):e1003790. doi: 10.1371/journal.pmed.1003790 (PMC8480908; doi:10.1371/journal.pmed.1003790)
Supplement: S3 Table — AMI, acute myocardial infarction; CI, confidence interval. (DOCX) [file pmed.1003790.s007.docx]

**S3 Table. Relative risks and 95% confidence intervals for acute myocardial infarction shortly after the death of a child**

| **Hazard period** | **Number of parents who lost a child in the hazard period** | **Control period**  **Relative risk (95% confidence intervals)** | | | | | |
| --- | --- | --- | --- | --- | --- | --- | --- |
|  |  | **Matched-pair control period ⃰** | **P-value** | **30-180 days prior to the AMI case** | **P-value** | **30-365 days prior to the AMI case** | **P-value** |
| 0-1 day | 7 | -† | - | 5.74 (2.66-12.39) | <0.001 | 5.61 (2.64-11.90) | <0.001 |
| 2-7 days | 5 | 1.25 (0.34-4.66) | 0.739 | 1.37 (0.56-3.37) | 0.494 | 1.34 (0.55-3.24) | 0.524 |
| 0-7 days | 12 | 2.99 (0.97-9.28) | 0.057 | 2.47 (1.35-4.50) | 0.003 | 2.41 (1.35-4.30) | 0.003 |
| 0-30 days | 22 | 1.22 (0.66-2.28) | 0.528 | 1.17 (0.74-1.87) | 0.498 | 1.14 (0.74-1.77) | 0.549 |

*The matched-pair control period was the same day of the week, week of the month or month of the year prior to the hazard period.

†There were no patients with acute myocardial infarction who experienced the death of their child 7-8 days prior to their acute myocardial infarction.
