# Supplementary material for: Death of an offspring and parental risk of ischemic heart diseases: A population-based cohort study
Source: PLoS Med. 2021 Sep 29;18(9):e1003790. doi: 10.1371/journal.pmed.1003790 (PMC8480908; doi:10.1371/journal.pmed.1003790)
Supplement: S4 Table — CI, confidence interval; IHD, ischemic heart disease; IRR, incidence rate ratio. (DOCX) [file pmed.1003790.s008.docx]

**S4 Table. Adjusted incidence rate ratios and 95% confidence intervals for the association between the death of a child and the risk of ischemic heart diseases in sensitivity analyses**

| **Study participants** | **Sensitivity analyses** | **N** | **IHD** | | **AMI** | |
| --- | --- | --- | --- | --- | --- | --- |
|  |  |  | **Multivariable IRR (95% CI)** | **P-value** | **Multivariable IRR (95% CI)** | **P-value** |
| All study participants | Multiple imputation for the missing data on education | 6,711,952 | 1.21 (1.18-1.24) | <0.001 | 1.21 (1.17-1.25) | <0.001 |
|  | Multiple imputation for missing data on education, marital status, income, parents’ and siblings’ history of CVD | 6,711,952 | 1.21 (1.18-1.24) | <0.001 | 1.21 (1.17-1.25) | <0.001 |
| Participants who did not lose a child prior to baseline | Main model 1 | 6,675,121 | 1.20 (1.18-1.23) | <0.001 | 1.20 (1.16-1.24) | <0.001 |
| Mothers with information on pregestational and gestational hypertension and diabetes at baseline⃰ | Main model 2 | 3,554,901 | 1.29 (1.24-1.34) | <0.001 | 1.35 (1.27-1.44) | <0.001 |
|  | Main model 2 + pregestational and gestational hypertension and diabetes | 3,554,901 | 1.29 (1.24-1.34) | <0.001 | 1.35 (1.27-1.43) | <0.001 |
| Mothers with information on maternal smoking in early pregnancy at baseline† | Main model 2 | 2,080,379 | 1.31 (1.18-1.45) | <0.001 | 1.42 (1.22-1.66) | <0.001 |
|  | Main model 2 + maternal smoking in early pregnancy | 2,080,379 | 1.25 (1.13-1.39) | <0.001 | 1.33 (1.14-1.55) | <0.001 |
| Mothers with information on maternal weight and height in early pregnancy at baseline‡ | Main model 2 | 1,907,015 | 1.22 (1.06-1.40) | 0.006 | 1.49 (1.23-1.80) | <0.001 |
|  | Main model 2 + maternal obesity in early pregnancy | 1,907,015 | 1.21 (1.05-1.39) | 0.009 | 1.47 (1.22-1.78) | <0.001 |

IRR=incidence rate ratio; CI=confidence intervals; IHD=ischemic heart disease; AMI=acute myocardial infarction; CVD=cardiovascular diseases.

In main model 1 we adjusted for sex, age at follow-up, calendar year at follow-up, country of birth, educational attainment, history of psychiatric disorders and of cardiovascular diseases.

In main model 2 we adjusted for age at follow-up, calendar year at follow-up, country of birth, educational attainment, history of psychiatric disorders and of cardiovascular diseases.

* Mothers who immigrated to Denmark or Sweden with children entered the cohort on the date of immigration and consequently lacked information on pregestational and gestational hypertension and diabetes at baseline.

† Information on maternal smoking in early pregnancy was available in the Medical Birth Register since 1997 in Denmark and since 1982 in Sweden.

‡Information on maternal weight and height in early pregnancy was available in the Medical Birth Register since 2003 in Denmark; information on maternal weight was available during 1982-1989 and 1992-2014 and on maternal height since 1982 in Sweden.
