# Supplementary material for: Death of an offspring and parental risk of ischemic heart diseases: A population-based cohort study
Source: PLoS Med. 2021 Sep 29;18(9):e1003790. doi: 10.1371/journal.pmed.1003790 (PMC8480908; doi:10.1371/journal.pmed.1003790)
Supplement: Attachment — Submitted filename: Response letter.docx [file pmed.1003790.s009.docx]

**Response to the editor’s and the reviewers’ comments**

**Manuscript’s ID:** PMEDICINE-D-21-00668R1

**Title:** Death of an offspring and parental risk of ischemic heart diseases: A population-based cohort study

Dear Dr. Richard Turner,

Thank you very much for the possibility to submit a revised version of our manuscript entitled “Death of an offspring and parental risk of ischemic heart diseases: A population-based cohort study”. We very much appreciate the editor’s and the reviewers’ constructive comments. Below we respond one by one to each comment raised and present the corresponding changes in the manuscript.

We hope that you will find the revised version of the manuscript acceptable for publication.

Sincerely yours,

Dang Wei, on behalf of all authors

Department of Global Public Health, Karolinska Institutet,

Solnavägen 1E, 113 65 Stockholm, Sweden

Email: dang.wei@ki.se

Tel: +46-738761749

**Editors:**

**Comment 1:**

*To your data statement (submission form), please add web addresses for the relevant data custodians for readers interested in inquiring about access to study data.*

**Response:**

In the submission form, we have now added the web addresses of the data holder authorities to the statement concerning data availability.

***Data availability section [submission form]***

All the data used in the present study were obtained from the Swedish National Board of Health and Welfare (<https://www.socialstyrelsen.se/en/about-us/contact-us/>), Statistics Sweden (https://www.scb.se), and Statistics Denmark (<https://www.dst.dk/en/kontakt>).

**Comment 2:**

*Please combine the "Methods" and "results" subsections of your abstract.*

**Response:**

We have now combined the “Methods” and the “Results” subsections of the abstract [Page 2, line 6-18].

**Comment 3:**

*The final sentence of the new combined subsection should begin "Study limitations include ..." or similar and should quote 2-3 of the study's main limitations.*

**Response:**

We now mention the study’s main limitations at the end of the section “Methods and findings”.

***Abstract section [Page 2, line 16-18]***

Study limitations include the possibility of residual confounding by socioeconomic, lifestyle or health-related factors and the potentially limited generalizability of our findings outside Scandinavia.

**Comment 4:**

*In the abstract and throughout the text, please include p values alongside 95% CI, where available.*

**Response:**

We have added p-values alongside 95% confidence intervals in both the abstract and in the manuscript’s main text.

**Comment 5:**

*Please remove the information on funding, competing interests and data access from between abstract and Introduction. In the event of publication, this information will appear only in the article metadata, via entries in the submission form.*

**Response:**

We have removed this information from the manuscript file.

**Comment 6:**

*After the abstract, please add a new and accessible "Author summary" section in non-identical prose. You may find it helpful to consult one or two recent research papers published in PLOS Medicine to get a sense of the preferred style.*

**Response:**

We have added an Author summary after the abstract.

***Author summary section [Page 3, line 1-24; Page 4, line 1-3]***

**Author summary**

**Why was this study done?**

- The death of a child is an extremely stressful life event, with potential consequences for parents’ cardiovascular health. Knowledge about the association between the loss of an offspring and the risk of ischemic heart diseases (IHD) and acute myocardial infarction (AMI), however, is very limited.
- If our hypotheses regarding such associations are correct, our findings may call for support from family, friends and health professionals and for increased surveillance for IHD for bereaved parents.

**What did the researchers do and find?**

- We conducted a population-based cohort study including 6.7 million parents from Denmark and Sweden to investigate the association between loss of an offspring and the parents’ risk of IHD and AMI.
- We found that parents who lost a child had higher risks of IHD and AMI than their unexposed counterparts. The associations were present not only if the offspring died due to cardiovascular and other natural causes, but also in case of unnatural deaths.
- The risk of AMI among bereaved parents was more than three times higher in the week after the death of the child and around 20% higher on the long term compared to that among non-bereaved parents.

**What do these findings mean?**

- The findings that the association was present also in case of losses due to unnatural causes, which are less likely to be confounded by cardiovascular risk factors clustering in families, and that the risk of AMI was highest during the week after the loss suggest that stress-related mechanisms may contribute to the observed associations.
- If confirmed in other studies, our findings may call for support from family, friends and health professionals and for increased surveillance for IHD for bereaved parents, in particular in the first week after the loss.

**Comment 7:**

*Early in the Methods section of your main text, please state whether the study had a protocol or prespecified analysis plan, and if so attach the relevant document(s) as an attachment(s), referred to in the text.*

*Please highlight analyses that were not prespecified.*

**Response:**

We now mention in the Methods section that we had a prespecified analyses plan and attach it. In the analysis plan, we have included a section containing the analyses that were suggested (1) by the coauthors during the drafting of the manuscript or (2) by the editors and reviewers of PloS Medicine during the revision of the manuscript.

***Methods section [Page 7, line 14-15]***

Our prespecified analysis plan is presented in the “Supporting information” (S1 Analysis plan).

**Comment 8:**

*On p.8, please substitute "sex" for "gender" if appropriate, and at any other points in the ms.*

**Response:**

We have now replaced “sex” with “gender” throughout the manuscript.

**Comment 9:**

*Throughout the text, please adapt reference call-outs to the following style: "... following the loss [1,3]." (noting the absence of spaces within the square brackets).*

*Please use the journal name abbreviation "PLoS ONE" in your reference list.*

**Response:**

We have now changed the format of the references according to your suggestion and use the abbreviation “PLoS ONE” in the reference list.

**Comment 10:**

*Please ensure that reference 29 and any others needing them have full access details.*

**Response:**

We have updated reference 29 (reference 31 in the revision) accordingly.

**Comment 11:**

*Please add a completed checklist for the most appropriate reporting guideline, e.g., STROBE, as an attachment, labelled "S1_STROBE_Checklist" and referred to as such in your Methods section.*

*In the checklist, please refer to individual items by section (e.g., "Methods") and paragraph number, not by line or page numbers as these generally change in the event of publication.*

**Response:**

We have uploaded a STROBE checklist prepared according to your instructions.

***Methods section [Page 7, line 15-17]***

We followed the Strengthening the Reporting of Observational Studies in Epidemiology guidelines when writing the manuscript (S1 STROBE Checklist).

**Reviewer #1:**

**Comment:**

*I confine my remarks to statistical aspects of this paper. These were very well done and I have only one issue to resolve before I can recommend publication.*

*The authors have an entire population. This makes the use of confidence intervals a bit troublesome. CIs are about inference from a sample to a population. When you have the whole population, there is no inference to be done and the CI should all be 0 width.*

*There are two options: The authors could delete the CI and not the above. On the other hand, some statisticians posit the existence of a "super-population" from which this population is randomly drawn (e.g. more countries, a longer time span, or something). I am not a big fan of this, but I wont object if the authors choose to go this route. But the issue should be addressed.*

*Otherwise - excellent job!*

**Response:**

Thank you very much for your positive review and for your suggestion. We agree that the relevance of confidence intervals is limited when an entire population is studied. However, our study population was restricted to parents with at least one live-born child during 1973-2016 in Denmark and during 1973-2014 in Sweden. To infer our findings to parents whose children were born before 1973 or whose first child was born after the end of our study period, confidence intervals might be helpful. Confidence intervals can also be considered to infer our findings to parents outside the studied countries. We now discuss the relevance of confidence intervals in the “Statistical analyses” section.

***Methods section [Page 9, line 10-14]***

We used Poisson regression to estimate incidence rate ratios (IRR) for the association between the death of a child and parental risk of incident IHD and AMI. Although we included almost all parents who gave birth to at least a child in Denmark or Sweden during our study period, we also calculated 95% confidence intervals (CI) to infer our findings to a theoretical larger group of potential parents.

**Reviewer #2:**

**Comment:**

*The authors submitted an interesting Research Article aiming at investigating whether the death of an offspring is associated with the risk of ischemic heart disease (IHD) and acute miocardici infarction (AMI). They found a significant association in case of losses due to CVD, or other natural causes, and also in case of unnatural deaths. So they concluded that the death of an offspring was associated with an increased risk of IHD and AMI. There's findings suggests that stress-related mechanisms may also contribute to the observed associations.*

*The manuscript is well written, even if more attention should be paid to English grammar and structure. Several typos are present through the whole manuscript and should be addressed before any acceptance for publication. The Authors should be commended for this tremendous work but some statistical issues should be refined in order to achieve more clarity and soundness. I recommend to show all the P-Values that here are missing in the text and the tables. A comparison between baseline characteristics between groups is also advisable, and than an adjustment for baseline statistical significant differences between groups will also helpful to better understand if many confounded have been addressed or not.*

**Response:**

Thank you very much for your positive review and for your constructive suggestions. We have now revised the manuscript for language and structure and have added all relevant *P*-values in the abstract, manuscript text and supplementary tables; we have also included in Table 1 a column with *P*-values corresponding to Student t-tests in case of continuous variables and chi-square tests in case of categorical variables. We now also specify on page 10 that, as recommended by Rothman K.J. (Rothman KJ, Greenland S, Lash TL. Modern Epidemiology, 3rd Edition: Lippincott Williams & Wilkins; 2008.), our criteria for including confounders in our multivariate models were (1) a known or an a priori considered plausible association with the death of a child and the risk of ischemic heart disease and acute myocardial infarction and (2) not being on the pathway between exposure and the outcome.

***Methods section [Page 9, line 8-10]***

We compared baseline characteristics of the exposed and the unexposed groups by means of Student t-tests in case of continuous variables and chi-square tests in case of categorical variables.

**Reviewer #3:**

**Comment 1:**

*This major study seeks to establish whether the death of an offspring is associated with the risk of IHD and AMI in Denmark (1973-2016) Sweden in the period 1973-2014 in the respective birth and linked health registers. 126,522 (1.9%) parents lost at least one child during the study period. The secondary outcome was 'unnatural deaths'. A total of >6.5M parents were followed. The comparison exposure group was non-bereaved parents. Parental fatal or nonfatal disease was determined in national registers. A large set of ICD codes was utilised, ranging from hypertension, diabetes to sudden coronary death. Major potential confounders of the association between parent-child bereavement are low income, low education and raised CVD risk including history of CVD. Such covariates were available in 80-95% of the population data, with gaps, e.g. little data on phenotypic CVD risk except in pregnancy. Multiple imputation could be utilized to fill in the missing data.*

**Response:**

Thank you very much for your positive review and for your constructive suggestions. We studied IHD and AMI as outcomes. “Unnatural deaths” referred to children. We now better clarify that the reason for performing analyses with exposure classified according to the cause of the child’s death (death due to cardiovascular diseases, other natural deaths, and unnatural deaths) was to contribute to a better understanding of the causality of the studied association. Compared with deaths due to cardiovascular diseases or other natural causes, unnatural deaths are less likely to be affected by cardiovascular risk factors that cluster in families; thus, compared to natural deaths, the association of unnatural deaths with the risk of IHD or AMI is less likely to be confounded by familial cardiovascular risk factors.

We imputed missing data by five replications using the fully conditional specification with logistic regression. After multiple imputation for the missing data, we re-ran our main model, i.e., adjusted for age (split at five years) and calendar year at follow-up (split at ten years) as time-varying variables and sex, country of birth, highest education, and history of psychiatric disorders and of CVD (model 2) and a maximally adjusted model, i.e., adjusted for marital status, income, parents’ history of CVD and siblings’ history of CVD in addition to variables in model 2 (model 3). We did not find any substantial changes in point estimates in the analyses with multiple imputation compared to those observed in the main analyses

***Methods section [Page 10, line 17-20]***

To address concerns about missing data for some covariates, we imputed missing data by five replications through the fully conditional specification using logistic regression [25]. After the multiple imputation for the missing data, we re-ran models 2 and 3.

***Results section [Page 11, line 20-24]***

The associations between the death of a child and the risk of IHD and AMI did not change substantially after 1) excluding study participants who lost a child before baseline, 2) controlling for pregestational and gestational hypertension and diabetes and maternal obesity in early pregnancy among women; the associations were slightly attenuated after adjusting for maternal smoking in early pregnancy, or 3) multiple imputation for missing data (S4 Table).

**Comment 2:**

*There would be increased clarity in this reviewer's reading for covariate stratified results to be presented second, in table 3 rather than 2 as at present. Table 2 could then focus on attenuation of the bereavement effect by adjustment for (1) the main a priori potential confounders, then (2) a further maximally adjusted model. It is unclear why the analysis is stratified according to IHD/AMI (tables 2 and 3). There does not seem to be a clear related hypothesis, and though there some marginally significant differences, the size of effects is so similar the stratification detracts from the paper and should be confined to sensitivity analyses. If the authors wish to hypothesise a specific link between bereavement and AMI then it should be more clearly articulated and analysed, comparing the time course of effect size with other outcomes.*

**Response:**

In Table 2, we now present three types of models: (1) age-adjusted models, (2) models including confounders for which we had data for the great majority of the study participants and (3) models including – in addition to variables in model 2 – also confounders with a large percentage of missing data. We now specify that our criteria for including confounders in our multivariable models were (1) a known or an a priori considered plausible association with the death of a child and the risk of ischemic heart disease and acute myocardial infarction and (2) not being on the causal pathway between exposure and the outcome. The stratified analyses according to sex, age, country, and education aimed to identify groups of bereaved parents at particularly increased risk to develop IHD/AMI. Therefore, we presented the results of the association of interest in Table 2 and those of the stratified analyses in Table 3, as suggested.

We performed analyses for any IHD and AMI separately due to the following reasons:

1) Although AMI is a subtype of IHD, it is the most severe acute manifestation of IHD and is a life-threatening condition. Several earlier studies have shown that bereavement may have a triggering, as well as a long-term effect on the risk of AMI. Better knowledge about the association between the death of a child and parent’s risk of AMI and the identification of the periods at highest risk may have implications for surveillance for cardiac symptoms during these periods. Furthermore, the validity of the AMI diagnosis is likely to be higher than that of overall IHD due to its life-threatening and emergent nature and the widely available and reliable diagnostic tools.
2) Any IHD consists of a broader spectrum of heart diseases and may represent a broader public health concern than AMI. With modern medical technology, IHD can now be detected in its early stages. Finding an association between the death of a child and an increased risk of IHD in parents may draw bereaved parents’ and professionals’ attention to the importance of early diagnosis and treatment of certain IHDs.

We now clarify the importance of investigating the association between the death of a parent and the risk of both IHD and AMI in the “Introduction” section.

**[The changes regarding the further maximally adjusted model]**

***Methods section [Page 9, line 17-25]***

We performed three type of models: model 1, adjusted for age at follow-up (split at five years), model 2, adjusted for age (split at five years) and calendar year at follow-up (split at ten years) as time-varying variables and sex, country of birth, highest education, and history of psychiatric disorders and of CVD as time-fixed variables, while model 3, adjusted for marital status, income, parents’ history of CVD and siblings’ history of CVD (i.e., confounders with a high rate of missing data) in addition to confounders in model 2. Criteria for including confounders in our multivariable models were 1) a known or an a priori considered plausible association with the death of a child and the risk of IHD and AMI and 2) not being on the pathway between exposure and the outcome [25].

***Table 2 [Page 23-24]***

**Table 2. Adjusted incidence rate ratios and 95% confidence intervals for ischemic heart disease according to the death of a child**

| **Exposure** | **Ischemic heart disease** | | | | | | | **Acute myocardial infarction** | | | | | | |
| --- | --- | --- | --- | --- | --- | --- | --- | --- | --- | --- | --- | --- | --- | --- |
|  | **Rate⃰** | **Model 1† (N=6,711,952)** | | **Model 2‡ (N=6,388,629)** | | **Model 3⁋ (N=4,512,391)** | | **Rate⃰** | **Model 1† (N=6,711,952)** | | **Model 2‡ (N=6,388,629)** | | **Model 3⁋ (N=4,512,391)** | |
|  |  | **IRR (95%CI)** | ***P*** | **IRR (95%CI)** | ***P*** | **IRR (95%CI)** | ***P*** |  | **IRR (95%CI)** | ***P*** | **IRR (95%CI)** | ***P*** | **IRR (95%CI)** | ***P*** |
| **Unexposed** | 222.5 | 1.00 |  | 1.00 |  | 1.00 |  | 108.7 | 1.00 |  | 1.00 |  | 1.00 |  |
| **All deaths** | 418.4 | 1.21  (1.18-1.24) | <0.001 | 1.20  (1.18-1.23) | <0.001 | 1.21  (1.17-1.26) | <0.001 | 205.7 | 1.18  (1.15-1.22) | <0.001 | 1.21  (1.17-1.25) | <0.001 | 1.22  (1.16-1.28) | <0.001 |
| **Cause of the child’s death** | | | | | | | | | | | | | | |
| Death due to CVD | 688.3 | 1.29  (1.17-1.42) | <0.001 | 1.32  (1.20-1.46) | <0.001 | 1.28  (1.11-1.49) | <0.001 | 381.8 | 1.39  (1.22-1.58) | <0.001 | 1.45  (1.27-1.66) | <0.001 | 1.40  (1.16-1.68) | <0.001 |
| Other natural death | 375.8 | 1.22  (1.18-1.25) | <0.001 | 1.21  (1.18-1.25) | <0.001 | 1.20  (1.14-1.26) | <0.001 | 180.5 | 1.17  (1.12-1.21) | <0.001 | 1.18  (1.14-1.23) | <0.001 | 1.21  (1.13-1.30) | <0.001 |
| Unnatural death | 553.8 | 1.17  (1.12-1.23) | <0.001 | 1.17  (1.12-1.23) | <0.001 | 1.21  (1.15-1.28) | <0.001 | 281.3 | 1.19  (1.12-1.27) | <0.001 | 1.23  (1.15-1.31) | <0.001 | 1.21  (1.12-1.30) | <0.001 |
| **Age of the deceased child at loss** | | | | | | | | | | | | | | |
| ≤1 | 224.4 | 1.31  (1.25-1.37) | <0.001 | 1.28  (1.22-1.34) | <0.001 | 1.29  (1.20-1.40) | <0.001 | 101.3 | 1.23  (1.15-1.32) | <0.001 | 1.22  (1.14-1.31) | <0.001 | 1.26  (1.13-1.41) | <0.001 |
| 2-12 | 291.2 | 1.15  (1.07-1.23) | <0.001 | 1.12  (1.05-1.20) | <0.001 | 1.10  (0.98-1.24) | 0.115 | 133.3 | 1.08  (0.98-1.19) | 0.118 | 1.06  (0.97-1.17) | 0.213 | 1.14  (0.96-1.34) | 0.131 |
| 13-18 | 460.8 | 1.12  (1.04-1.21) | 0.003 | 1.11  (1.03-1.20) | 0.004 | 1.07  (0.96-1.21) | 0.228 | 213.2 | 1.06  (0.95-1.18) | 0.324 | 1.06  (0.95-1.18) | 0.285 | 1.07  (0.91-1.25) | 0.432 |
| 19-29 | 643.3 | 1.18  (1.14-1.23) | <0.001 | 1.18  (1.13-1.23) | <0.001 | 1.20  (1.13-1.27) | <0.001 | 318.6 | 1.18  (1.12-1.25) | <0.001 | 1.20  (1.13-1.27) | <0.001 | 1.17  (1.08-1.27) | <0.001 |
| >29 | 1035.1 | 1.22  (1.17-1.28) | <0.001 | 1.27  (1.21-1.34) | <0.001 | 1.27  (1.18-1.37) | <0.001 | 552.5 | 1.25  (1.18-1.34) | <0.001 | 1.35  (1.26-1.44) | <0.001 | 1.36  (1.24-1.49) | <0.001 |
| **Number of remaining live children at loss** | | | | | | | | | | | | | | |
| 0 | 228.2 | 1.19  (1.12-1.27) | <0.001 | 1.17  (1.10-1.25) | <0.001 | 1.24  (1.13-1.36) | <0.001 | 109.1 | 1.17  (1.07-1.28) | <0.001 | 1.17  (1.07-1.28) | <0.001 | 1.17  (1.02-1.34) | 0.026 |
| 1-2 | 417.4 | 1.15  (1.12-1.19) | <0.001 | 1.17  (1.14-1.21) | <0.001 | 1.17  (1.12-1.23) | <0.001 | 197.8 | 1.10  (1.05-1.15) | <0.001 | 1.14  (1.09-1.19) | <0.001 | 1.16  (1.09-1.24) | <0.001 |
| ≥3 | 722.7 | 1.37  (1.31-1.44) | <0.001 | 1.32  (1.26-1.38) | <0.001 | 1.28  (1.20-1.38) | <0.001 | 384.4 | 1.42  (1.34-1.51) | <0.001 | 1.40  (1.32-1.48) | <0.001 | 1.38  (1.26-1.50) | <0.001 |

IRR=incidence rate ratio; CI=confidence intervals; CVD=cardiovascular disease.

* Per 100,000 person-years.

† Model 1 adjusted for age at follow-up.

‡ Model 2 adjusted for gender, age at follow-up, calendar year at follow-up, country of birth, educational attainment, history of psychiatric disorders and of cardiovascular diseases at baseline.

⁋ Model 3 adjusted for gender, age at follow-up, calendar year at follow-up, country of birth, educational attainment, marital status at baseline, income at baseline, history of psychiatric disorders and of cardiovascular diseases at baseline, parents’ history of cardiovascular diseases, and siblings’ history of cardiovascular diseases.

**[The changes regarding analyses for IHD and AMI separately]**

***Introduction section [Page 5, line 22-25; Page 6, line 1-2]***

Given the low prevalence of exposure, the low incidence of atherosclerotic CVDs in early and mid-adulthood, and the observed modest association, the study of Li et al. did not have sufficient statistical power to analyze the importance of the type of death, potential effect modifiers of this association, nor short-term effects after the loss; a few earlier studies have suggested that the death of other type of family members may trigger AMI [5,8].

***Introduction section [Page 6, line 4-8]***

In addition to AMI, the death of a child may increase the risk of other, less severe ischemic heart diseases (IHDs) such as angina pectoris and atherosclerosis that may eventually lead to an AMI. Modern medical technology allows an early detection of IHD, with implications for the prevention of AMI. However, the association between the death of a child and the risk of overall IHDs has not yet been studied.

**Comment 3:**

*The Discussion addresses the main expected topics, including key findings, past studies, bias, power and mechanisms. The precise role of the unnatural causes of death as comparator outcome could be better articulated, particularly whether this new study strengthens or weakens the accumulated evidence for a specific link between bereavement and CVD. I would prefer to see a more focused use of the terms 'bereavement stress' and 'grief' including in the abstract. Use of the general term 'stress' is widespread and can lack scientific coherence. For example there may be short or extended emotional grief responses to bereavement, followed by depressed mood, sadness and so on. Cognitive appraisal of the personal impact and meaning of the bereavement could affect health behaviours, independent of 'stress mechanisms'.*

**Response:**

We used death due to unnatural causes of the child and not of the parent as a comparator. We emphasize in the Discussion that the reason for performing such an analysis is that the effect of deaths due to unnatural causes on the risk of IHD or AMI is less likely to be affected by confounding due to cardiovascular risk factors that cluster in families. We now use the terms “bereavement stress” and “grief” instead of “stress” whenever possible throughout the text.

***Discussion section [Page 12, line 2-5]***

To try to separate the effect of grief after the death of the child from that of confounding by cardiovascular risk factors that cluster in families, we performed analyses with exposure classified according to the child’s cause of death.

***Discussion section [Page 14, line 20-24]***

Bereavement stress stimulates the activation of the hypothalamic-pituitary-adrenocortical axis and of the sympathetic nervous system, which in turn leads to a short-term increased inflammatory activity, elevated cortisol levels, total cholesterol, blood pressure and heart rate and reduction of heart rate variability and high-density lipoprotein [1,37-39], which in turn could trigger AMI [40].

***Discussion section [Page 15, line 6-9]***

Chronic bereavement stress and complicated grief may induce adverse changes in health behaviours and in endocrine, immune, vascular and haemostatic activities contributing to further progression of atherosclerosis and thus increasing the risk of IHD and AMI.
