# Supplementary material for: Death of an offspring and parental risk of ischemic heart diseases: A population-based cohort study
Source: PLoS Med. 2021 Sep 29;18(9):e1003790. doi: 10.1371/journal.pmed.1003790 (PMC8480908; doi:10.1371/journal.pmed.1003790)
Supplement: Attachment — Submitted filename: Response letter_revision 2_20210831.docx [file pmed.1003790.s010.docx]

**Response to the editor’s and the reviewers’ comments**

**Manuscript’s ID:** PMEDICINE-D-21-00668R2

**Title:** Death of an offspring and parental risk of ischemic heart diseases: A population-based cohort study

Dear Dr. Richard Turner,

Thank you very much for provisionally accepting our manuscript entitled “Death of an offspring and parental risk of ischemic heart diseases: A population-based cohort study” for publication in PLOS Medicine. We very much appreciate the editor’s and the reviewers’ positive comments. Below we respond one by one to each comment raised and present the corresponding changes in the manuscript.

We hope that you will find the revised version of the manuscript ready for publication.

Sincerely yours,

Dang Wei, on behalf of all authors

Department of Global Public Health, Karolinska Institutet,

Solnavägen 1E, 113 65 Stockholm, Sweden

Email: dang.wei@ki.se

Tel: +46-738761749

**Editors:**

**Comment 1:**

*Please cite your recent paper (doi: 10.1093/eurheartj/ehaa1084) at an appropriate point in the paper (we became aware of this paper through an iThenticate check).*

**Response:**

We have now cited our recent paper published in European Heart Journal in several places in the manuscript, i.e. on pages 6, line 8; page 6, line 15; page 7, line 18; page 11, line 5 and 19; page 13, line 14; page 16, line 2.

**Comment 2:**

*Please quote summary demographic details for study participants in the abstract.*

**Response:**

We now present in the abstract the mean age at baseline and the gender distribution of the cohort.

***Abstract section [Page 2, line 6-8]***

We studied parents of live-born children recorded in the Danish (1973-2016) and the Swedish (1973-2014) Medical Birth Registers (n=6,711,952; mean age at baseline 31 years, 53% women).

**Comment 3:**

*At line 16 in the abstract please make that "follow-up period".*

**Response:**

We have changed “follow-up” to “follow-up period”, as suggested.

**Comment 4:**

*In the Summary points, the wording of the second and final point is very similar, and we ask you to amend these points to make them less repetitive.*

**Response:**

We have modified the second summary point based on your suggestion; it now runs “If our hypotheses regarding such associations are correct, this knowledge may raise awareness about the importance of support in bereavement and of increased cardiovascular risk monitoring for bereaved parents.”

**Comment 5:**

*Under "Strengths and limitations" in the Discussion section you mention the "... prospective design" as a strength, and we ask you to remove the word "prospective". You might wish to mention your prespecified analysis plan as a strength instead.*

**Response:**

We have now removed the word “prospective” and have mentioned the prespecified analysis plan as a strength.

***Discussion section [Page 16, line 18-21]***

Several strengths of our study make the findings robust, i.e. the prespecified analysis plan, the population-based design, the collection of information on exposure and outcome independently of each other, and the high quality information on mortality and the diagnoses of IHD and AMI in the two patient registers [43,44].

**Comment 6:**

*Please substitute "sex" in place of "gender" where appropriate (e.g., in table 1).*

**Response:**

We have now replaced “gender” by “sex” throughout the manuscript.

**Comment 7:**

*Please spell out the institutional author name for reference 12.*

**Response:**

We have now re-formatted reference 12 (reference 13 in the revision) to spell out the institutional author name.

**Reviewer #1:**

**Comment:**

*The authors have addressed my concerns and I now recommend publication.*

**Response:**

Thank you very much for your positive comments.

**Reviewer #2:**

**Comment:**

*The manuscript significantly improved after revisions. I have no further comments or edits.*

**Response:**

Thank you very much for your positive comments.
